# Mapping quantitative trait loci for biomass yield and yield-related traits in lowland switchgrass (*Panicum virgatum* L.) multiple populations

**DOI:** 10.1093/g3journal/jkad061

**Published:** 2023-03-22

**Authors:** Surya L Shrestha, Christian M Tobias, Hem S Bhandari, Jennifer Bragg, Santosh Nayak, Ken Goddard, Fred Allen

**Affiliations:** Department of Plant Sciences, University of Tennessee, 112 Plant Biotechnology Building, Knoxville, TN 37996-4500, USA; United States Department of Agriculture (USDA) Agricultural Research Service (ARS), Western Regional Research Center, 800 Buchanan Street, Albany, CA 94710, USA; Plant Systems-Production, USDA National Institute of Food and Agriculture (NIFA), Beacon Complex, USA; Department of Plant Sciences, University of Tennessee, 112 Plant Biotechnology Building, Knoxville, TN 37996-4500, USA; United States Department of Agriculture (USDA) Agricultural Research Service (ARS), Western Regional Research Center, 800 Buchanan Street, Albany, CA 94710, USA; Department of Plant Sciences, University of Tennessee, 112 Plant Biotechnology Building, Knoxville, TN 37996-4500, USA; USDA ARS, Crop Improvement and Protection Research Unit, 1636 E Alisal Street, Salinas, CA 93905, USA; Department of Plant Sciences, University of Tennessee, 112 Plant Biotechnology Building, Knoxville, TN 37996-4500, USA; Department of Plant Sciences, University of Tennessee, 112 Plant Biotechnology Building, Knoxville, TN 37996-4500, USA

**Keywords:** bioenergy, biomass, environment, genome, hybrid, phenotype, plant genetics and genomics

## Abstract

Switchgrass can be used as an alternative source for bioenergy production. Many breeding programs focus on the genetic improvement of switchgrass for increasing biomass yield. Quantitative trait loci (QTL) mapping can help to discover marker-trait associations and accelerate the breeding process through marker-assisted selection. To identify significant QTL, this study mapped 7 hybrid populations and one combined of 2 hybrid populations (30–96 F1s) derived from Alamo and Kanlow genotypes. The populations were evaluated for biomass yield, plant height, and crown size in a simulated-sward plot with 2 replications at 2 locations in Tennessee from 2019 to 2021. The populations showed significant genetic variation for the evaluated traits and exhibited transgressive segregation. The 17,251 single nucleotide polymorphisms (SNPs) generated through genotyping-by-sequencing (GBS) were used to construct a linkage map using a fast algorithm for multiple outbred families. The linkage map spanned 1,941 cM with an average interval of 0.11 cM between SNPs. The QTL analysis was performed on evaluated traits for each and across environments (year and location) that identified 5 QTL for biomass yield (logarithm of the odds, LOD 3.12–4.34), 4 QTL for plant height (LOD 3.01–5.64), and 7 QTL for crown size (LOD 3.0–4.46) (*P* ≤ 0.05). The major QTL for biomass yield, plant height, and crown size resided on chromosomes 8N, 6N, and 8K explained phenotypic variations of 5.6, 5.1, and 6.6%, respectively. SNPs linked to QTL could be incorporated into marker-assisted breeding to maximize the selection gain in switchgrass breeding.

## Introduction

Use of molecular markers can improve genetic gains and assist in the faster development of new cultivars through marker-assisted breeding (Jiang 2013). Identifying genetic markers associated with traits is critical for successfully incorporating marker-assisted selection in plant breeding. Due to high abundance in the genome and ease of scoring, single nucleotide polymorphisms (SNPs) have become prevalent in genetics and breeding applications (Chen *et al*. 2014). Genotyping by sequencing (GBS) allows higher sequence coverage of specific loci by initiating sequencing reads from restriction endonuclease recognition sites and thus allows reliable SNP calling in a cost-effective manner (Elshire *et al*. 2011). The GBS approach has been successfully employed on barley and wheat (Poland *et al*. 2012) and switchgrass (Fiedler *et al*. 2015) for generating high-density linkage maps. With the advancement of a reference genome assembly of switchgrass (Lovell *et al.* 2021), the alignment of molecular markers to genome coordinates has allowed both physical and genetic distances to be determined with some precision (Dong *et al*. 2015; Bragg *et al*. 2020). Several studies have performed linkage analyses of quantitative traits in switchgrass. Chang *et al*. (2016) identified 26 QTL for tillering traits in both self and hybrid populations, and the evaluated traits had a good correlation with biomass yield (BMY). Using a full-sib mapping population derived from a cross between lowland cultivars (Alamo and Kanlow), Lowry *et al*. (2015) identified 27 QTL for growth, morphological, and phenological traits. Four QTL for BMY and 5 QTL for plant height (PHT) explaining phenotypic variation up to 12% were mapped on a heterozygous pseudo-F1 population derived from a cross between lowland (Alamo) and upland (Summer) genotypes (Serba *et al*. 2015).

Many studies have demonstrated the existence of genotypic variation for BMY in both lowland and upland switchgrass (Serba *et al*. 2015; Shrestha *et al*. 2021). Some studies have focused on dissecting the complexity of BMY through markers associated with secondary or component yield traits (Lowry *et al*. 2015; Chang *et al*. 2016; Ali *et al*. 2019). Serba *et al*. (2015) mapped several QTL for BMY and PHT in F1 populations derived from the cross of upland and lowland ecotypes. High planting density (30 cm between plants) was used in our study, which helped to better estimate the genetic effects as compared to Serba *et al*. (2015), in which genotypes were evaluated in low planting density (150 cm between plants). Whether or not the same loci contribute to BMY variation in lowland × lowland crosses is an important question.

Multiple segregating F1 populations with different genetic backgrounds have many advantages over the single biparental population. In the QTL mapping of multiple segregating populations, multiple alleles are considered at a time, increasing the power of QTL detection (Buckler *et al*. 2009; Reif *et al*. 2011; Würschum *et al*. 2012). Also, the accuracy of QTL localization and the efficacy of QTL effects is higher in multiple segregating populations than in a single biparental population (Liu *et al*. 2012). Many QTL have been identified in a variety of crops, including maize (Buckler *et al*. 2009), wheat (Reif *et al*. 2011), and rapeseed (Würschum *et al*. 2012), using multiple populations.

The objective of this study was to identify genomic regions associated with BMY and yield-related traits, including PHT and crown size (CRS), across multiple hybrid (F1) populations produced within crosses between switchgrass cultivars Alamo and Kanlow. Shrestha *et al*. (2021) found significant variations in BMY, PHT, and CRS in hybrid populations of these cultivars that were likely controlled by many genes. Identifying genetic markers associated with these traits could improve selection efficiency through the introgression of these markers into germplasms using marker-assisted selection techniques.

## Materials and methods

### Mapping population

Nine F1 populations (30–96 F1 genotypes per population) were selected based on visual vigor, including biomass and PHT, from a set of 44 biparental crosses using individual Alamo (A) and Kanlow (K) derived sublines (Bhandari *et al*. 2017). Sets of F1 ramets (79–96) were derived from the remnant seeds of 8 of the selected populations. Each ramet was clonally propagated to produce enough seedlings to plant in a replicated field trial. The 8 populations include, 11A-88 × 12K-35 (*n* = 90), 12A-213 × 11K-233 (*n* = 84), 12A-221 × 12K-216 (*n* = 93), 12A-251 × 12K-217 (*n* = 90), 12A-259 × 12K-220 (*n* = 96), 12A-259 × 12K-247 (*n* = 90), 12A-261 × 12K-245 (*n* = 82), and 12A-263 × 12K-250 (*n* = 79), respectively. For the population TN13004–08 (A) × TN13009–08 (K) (*n* = 30), which did not have enough F1 seeds, the ramets were produced through the clones planted in the previous study (Bhandari *et al*. 2017). The selected populations exhibited varying BMY performance and yield heterosis (Bhandari *et al*. 2017; Shrestha *et al*. 2021).

### Field layout and experimental conditions

A set of genotypes of each of the 8 F1 populations were divided into 2 subsets, each with approximately equal numbers of plants, and planted at 2 locations in Tennessee: East Tennessee Research and Education Center (ETREC), Knoxville (270 m asl; 35.9006° *N*, −83.9554° W), and the Plateau Research and Education Center (PREC), Crossville (568 m asl; 36.0133° *N*, −85.1324° W). We did not have enough F1 seeds for each population to replicate in the locations, and we geographically split the population into half to evaluate any changes that might occur due to natural selection or by random chance. Therefore, the ramets of each population planted at the 2 locations were derived from different seedling genotypes. ETREC is located in hardiness Zone 7a (with an average low temperature of 0–5°C), and PREC is located in Zone 6b (with an average low temperature of −5 to 0°C). The PREC is slightly cooler with higher annual precipitation than the ETREC location. The cross TN13004-08 (A) × TN13009-08 (K) was evaluated only at the PREC site. Parental clones of the populations were evaluated in both locations. A randomized complete block design with 2 replications was used at each location. Six ramets of each genotype (per replication) were planted in a single row of the simulated-sward plot with a plant-to-plant spacing of 30 cm within a row and 120 cm between rows.

The field experiments were transplanted on 6 and 7 August 2018 at ETREC on a Huntington silt loam (fine loamy, mixed, sub-active, thermic, Typic Hapludults) and on 20 and 21 June 2018 at PREC on a Lily silt loam (fine loamy, siliceous, semi-active, mesic, Typic Hapludults) soil. The plots were amended with 60 kg *N* ha^−1^ in the spring of the establishment and postestablishment years (2019–2021). Preemergence herbicides, including Dual II Magnum (*S*-Metolachlor, Syngenta Crop Protection) at 2.84 L ha^−1^ and Prowl H_2_O (Pendimethalin, BASF Corporation) at 3.31 L ha^−1^ were applied at the time of planting and in the early spring of each of the years after establishment. Postemergence herbicide 2,4-dichlorophenoxyacetic acid (2,4-D) at 2.37 L ha^−1^ with surfactant at 1.18 L ha^−1^ was applied each spring.

### Assessment of phenotypic traits

PHT and BMY were measured at maturity, and CRS was evaluated after harvesting biomass. The measurements were taken in the fall of 2019, 2020, and 2021 (2-, 3-, and 4-years old stands) as the process described by Shrestha *et al*. (2021). PHT was measured in cm from the base of the plant to the tip of the panicle. The plots were harvested on 15 November in 2019, 6 and 9 November in 2020, and 18 and 19 October in 2021 at ETREC using a Carter flail forage harvester (Carter Manufacturing Company, Inc., Brookston, IN). At PREC, plots were harvested on 25 and 26 November in 2019, 4 November in 2020, and 21 and 22 October in 2021 using a Gehl forage chopper (Gehl Farm Equipment, West Bend, WI). BMY was measured by harvesting plants at 20 cm from the soil surface and weighing the plot biomass in bulk. An approximately 200 g sample of the harvested biomass was collected from each plot to determine the moisture content at harvest. The fresh weight of the sample was measured using a bench scale (American Scientific LLC, Columbus, OH), dried sample in a batch oven (Wisconsin Oven, East Troy, WI) at 48°C for 72 h, and reweighed to determine the dry weight (DW). Moisture percentage in the sample was determined as $100\times \left[\frac{FW-DW}{FW}\right]$. Plot yields were converted to megagrams per hectare (Mg ha^−1^) on a DW basis. CRS was scored on a score of 1–5, where a score of 1 indicates a low number of stems and a small diameter of the clone spread. The CRS of 4 center plants within each plot (6 plants per plot) was individually scored, and the mean value was calculated for each plot. A handheld electronic HarvestMaster (Juniper Systems, Inc., Logan, UT) was used to record BMY and other phenotypic data.

### Statistical analyses

Two-way analysis of variance across years for each location was performed using 3 years (2019–2021) and 2 locations (ETREC and PREC) data for BMY, PHT, and CRS. The data were analyzed using a mixed-effects model in JMP Pro 15 (SAS Institute), where genotype (F1 population or parent) and year were considered fixed effects, and replication within the year was considered a random effect. The mean distribution of measured traits was derived for each population in each year and location. Approximately 20% of the plots were lost at ETREC in 2019 and 2020 from a combination of animal damage and harvesting errors. Plots with 3 or more missing plants were considered missing plots for the analyses. Yield values of those plots with one or 2 plants missing were adjusted by adding to the initial plot weight, the average observed per plant yield in the plot, multiplied by the number of missing plants (1 or 2). The least-square mean (LS Mean) values were used to derive the relationships between traits using the linear regression (*R^2^*) test. The *R^2^* across populations was evaluated for each location (ETREC and PREC) across years (2019, 2020, and 2021) and across locations and years.

### Genotype analysis

Young leaf tissue was collected from each F1 progeny and parents and stored at −80 °C until processing for DNA extraction. The frozen leaves were lyophilized for 72 h in a Labconco freeze dryer (Labconco, Kansas City, MO), ground to a fine powder using a TissueLyser II (Qiagen, Valencia, CA), and DNA was extracted using the cetyltrimethylammonium bromide procedure. The extracted DNA was genotyped at the United States Department of Agriculture (USDA)-Agricultural Research Service (ARS) Western Regional Research Center laboratory in Albany, CA. Genotyping by sequencing was performed on 951 lines (F1s and their parents), and we obtained 4.3 ± 1.8 M reads per sample. The quality of these sequences showed that 94.4% of the bases were at or above the Phred quality score (Q30). Reads were mapped to version 5.0 of the switchgrass reference genome. SNP calling was performed using bwa (Li and Durbin 2009), bcftools (Li 2011), and samtools (Li 2011). A total of 4,678,008 sites were filtered down to 226,318 after setting genotypes with a depth of one to missing and filtering out sites that were missing in more than 10% of the lines. Further filtering to remove redundant markers and those with low frequency reduced the number of markers for linkage map construction (Supplementary Table 1).

### Construction of linkage map

A consensus linkage map was produced with Lep-Map3 software (Rastas 2017) from the combined 8 families using genotype probabilities of SNP that were informative in 6 or more families. Based on segregation analysis, one set of full-sibs that we had originally believed to be independent populations (12A-261 × 12K-245 and 12A-263 × 12K-250) possibly duplicated while making parental crosses were considered as a combined population for phenotypic and QTL analysis. Problematic individuals were removed from analysis based on kinship estimates, selfing detection, and unlikely recombination events. These filtering steps are summarized in Supplementary Table 1. SNP were then ordered, and the parental phase was estimated using the Lep-Map3 likelihood maximization method. The switchgrass reference genome was used to aid marker ordering, and the final genetic map is closely tied to the reference sequence. The final map was created based on linkage analysis of 648 individuals at a logarithm of the odds (LOD) value of 16.

### QTL mapping

For QTL mapping, the LS mean values of the 2 replicates of each genotype were calculated using data from each location and year. The LS mean of a genotype across locations was derived from the data across ETREC and PREC and across years was derived from the data across 2019, 2020, and 2021. QTL mapping across the population was performed for all measured traits, including BMY, PHT, and CRS, within each environment (location and year) and across the environments. A genome scan was performed using the scanone function in the R-QTL package (Broman *et al*. 2003). QTL detection for traits was based on a composite interval mapping using the Haley-Knott regression method (Broman and Sen 2009). The LOD threshold for each trait was calculated based on 1,000 permutations. An allele associated with the QTL was identified using genotype information. The significance of allele on QTL detection was tested using Tukey–Kramer Honest Significance Test (HSD; *P <* 0.05).

## Results

### Phenotypic variation

**Fig. 1.**
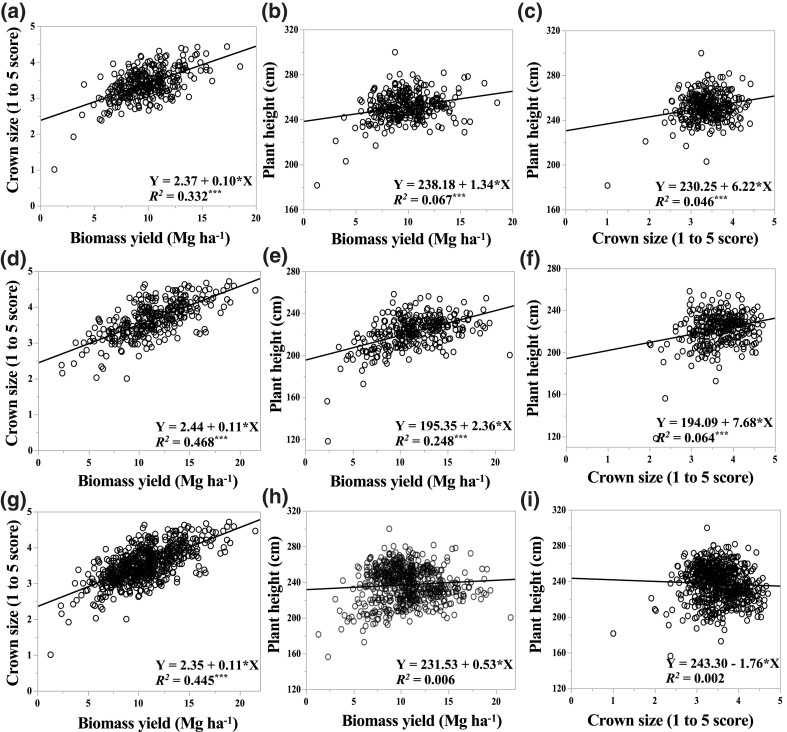
Relationships of biomass yield, plant height, and crown size across 8 Alamo and Kanlow populations at ETREC across years (2019–2021) (a–c), PREC across years (2019–2021) (d–f), and across locations (ETREC and PREC) and years (2019–2021) (g–i). *, **, ***: significant at *P* ≤ 0.05, *P*≤ 0.01, and *P*≤0.001, respectively.

The effect of genotype was significant for all crosses and traits (Table 1). The effect of the year was significant for all measured traits at ETREC but only for CRS at PREC. The interaction effect of genotypes and year was significant for BMY of population 12A-259 × 12K-247. The BMY of the populations varied from 8.8 to 11.1 Mg ha^−1^ at ETREC and 10.7 to 12.2 Mg ha^−1^ at PREC across the years (Table 2). Biomass yields were 6.7 Mg ha^−1^ higher at PREC than at ETREC. The PHT of the populations varied from 246 to 256 cm at ETREC and 215 to 231 cm at PREC across the years (Table 2). The CRS was similar in ETREC and PREC locations (Table 2). There was a positive association between BMY and CRS (*R^2^* = 0.33 and 0.47 at ETREC and PREC, respectively) (Fig. 1, a and d), BMY and PHT (*R^2^* = 0.07 and 0.25 at ETREC and PREC, respectively) (Fig. 1, b and e), and CRS and PHT (*R^2^* = 0.01 and 0.01 at ETREC and PREC, respectively) (Fig. 1, c and f) across years (2019–2021). CRS was found as a good predictor of BMY across locations and years (*R^2^* = 0.45) (Fig. 1g), whereas no significant association was observed between BMY and PHT and CRS and PHT across locations and years (Fig. 1, h and i).

**Table 1.** Sources of variation (SOV) of biomass yield, plant height, and crown size at the East Tennessee Research and Education Center (ETREC) and Plateau Research and Education Center (PREC) across 2019, 2020, and 2021.

F1 populations	SOV	ETREC, Knoxville, TN						PREC, Crossville, TN					
		Biomass yield		Plant height		Crown size		Biomass yield		Plant height		Crown size	
		df	F ratio	df	F ratio	df	F ratio	df	F ratio	df	F ratio	df	F ratio
11A-88 × 12K-35	Genotype (G)	38^b^	3.62***	40	3.72***	40	5.43***	41	5.0***	41	4.12***	41	3.43***
	Year (Y)	2	54.55**	2	9.29	2	41.15**	2	0.98	2	1.39	2	116.31**
	G × Y	76	0.93	80	0.73	80	0.84	82	0.61	82	0.59	82	0.77
12A-213 × 11K-233	G	34^e^	2.69***	40^a^	5.10***	41	3.50***	31	5.64***	31	4.45***	31	4.69***
	Y	2	303.13	2	35.2*	2	28.64*	2	0.69	2	0.49	2	31.75**
	G × Y	68	0.70	80	0.7	82	0.51	62	0.76	62	0.86	62	0.37
12A-221 × 12K-216	G	42^a^	2.62***	43	3.97***	43	2.49***	42	4.05***	42	5.69***	42	2.65***
	Y	2	1059.41**	2	6.037	2	85.15**	2	3.21	2	2.9	2	31.5**
	G × Y	84	0.92	86	0.68	86	0.65	84	0.54	84	0.59	84	0.79
12A-251 × 12K-217	G	39^c^	2.78***	41^a^	3.44***	42	2.34***	38	7.97***	38	5.04***	38	5.25***
	Y	2	635.90*	2	27.77*	2	35.69**	2	3.27	2	4.09	2	67.93**
	G × Y	78	1.04	82	0.82	84	0.64	76	0.73	76	1.11	76	0.66
12A-259 × 12K-220	G	38^c^	2.48***	40^a^	3.52***	41	3.03***	36	3.4***	36	3.52***	36	4.97***
	Y	2	161.88***	2	29.64**	2	171.54***	2	3.73	2	2.72	2	47.32**
	G × Y	76	0.55	80	0.8	82	0.27	72	0.49	72	0.56	72	0.9
12A-259 × 12K-247	G	37^a^	5.79***	38	5.63***	38	4.51***	34	8.23***	34	3.51***	34	5.36***
	Y	2	245.88*	2	60.55**	2	82.62**	2	9.02	2	3.63	2	17.3**
	G × Y	74	1.47*	76	1.05	76	0.5	68	0.74	68	0.67	68	0.57
12A-261 × 12K-245 and 12A-263 × 12K-250	G	62^d^	2.82***	65^a^	6.63***	65^a^	3.58***	43	5.43***	43	3.46***	43	3.79***
	Y	2	82.75**	2	15.56*	2	150.17**	2	0.12	2	1.26	2	113.02
	G × Y	124	0.75	130	0.8765	130	0.78	86	0.59	86	0.69	86	0.68
TN13004-08 (A) × TN13009-08 (K)	G	—	—	—	—	—	—	28^a^	8.32***	28^a^	4.52***	28^a^	4.84***
	Y	—	—	—	—	—	—	2	1.73	2	1.19	2	170.65**
	G × Y	—	—	—	—	—	—	56	0.66	56	0.69	56	0.77

df, degree of freedom; df^a,b,c,d^, and ^e^ indicates 1, 2, 3, 4, and 7 genotypes missing in the populations due to animal damage and sampling errors; “A” indicates the Alamo source parent, and “K” indicates the Kanlow source parent; “—”, no data available for the population at ETREC.

*, **, ***: significant at *P* ≤ 0.05, *P* ≤ 0.01, and *P*≤0.001, respectively.

**Table 2.** Mean, maximum (Max.), minimum (Min.), and standard error (SE) of biomass yield, plant height, and crown size on 7 individual and one combined Alamo (A) and Kanlow (K) populations and their parents at ETREC and PREC across 2019, 2020, and 2021.

Source material	ETREC, Knoxville, TN											
	Biomass yield (Mg ha^−1^)				Plant height (cm)				Crown size (1–5 score)			
	Mean	Max.	Min.	SE	Mean	Max.	Min.	SE	Mean	Max.	Min.	SE
11A-88 × 12K-35	9.11	20.12	1.55	0.41	256	305	206	1.5	3	5	2	0.05
12A-213 × 11K-233	8.84	19.15	0.65	0.4	246	292	180	1.74	3	5	1	0.06
12A-221 × 12K-216	9.18	26.43	1.60	0.43	251	300	211	1.27	3	5	2	0.05
12A-251 × 12K-217	9.34	25.61	0.67	0.43	250	305	198	1.45	3	5	2	0.04
12A-259 × 12K-220	11.07	27.04	1.60	0.52	248	305	211	1.12	4	5	2	0.06
12A-259 × 12K-247	8.94	23.42	1.20	0.44	254	305	193	1.65	3	5	1	0.05
12A-261 × 12K-245 and 12A-263 × 12K-250*^a^*	10.09	23.48	1.82	0.36	254	307	184	1.22	3	5	2	0.04
Alamo	8.15	16.97	1.68	1.04	245	274	216	4.01	3	4	3	0.11
Kanlow	6.47	12.28	1.66	0.76	255	279	234	3.03	3	4	2	0.1
	**PREC, Crossville, TN**											
11A-88 × 12K-35	10.94	20.01	2.74	0.32	226	268	170	1.53	4	5	3	0.05
12A-213 × 11K-233	10.48	26.15	1.36	0.43	219	264	138	1.98	3	5	2	0.07
12A-221 × 12K-216	10.71	20.64	5.37	0.22	223	267	184	1.37	4	5	2	0.05
12A-251 × 12K-217	11.32	24.60	3.70	0.36	217	249	149	1.63	4	5	2	0.06
12A-259 × 12K-220	12.15	22.73	4.82	0.33	224	260	189	1.4	4	5	2	0.06
12A-259 × 12K-247	12.13	20.72	2.95	0.38	231	267	173	1.59	4	5	2	0.07
12A-261 × 12K-245 and 12A-263 × 12K-250*^a^*	11.75	20.95	2.84	0.33	222	259	177	1.47	4	5	2	0.06
TN13004-08 (A) × TN13009-08 (K)	11.57	21.05	1.60	0.4	215	251	114	1.99	4	5	1	0.07
Alamo	7.3	11.43	2.62	0.52	201	229	152	3.97	3	5	1	0.20
Kanlow	7.49	10.97	4.93	0.36	220	253	174	3.47	3	5	3	0.12

Populations 12A-261 × 12K-245 and 12A-263 × 12K-250 were consolidated based on segregation analysis and considered a single cross for phenotypic and QTL analysis.

The average BMY across all populations was 30 and 55% higher than parents at ETREC and PREC (Table 2). A similar trend was observed for PHT and CRS. The 2 parents differed considerably in BMY and PHT (*P* ≤ 0.05) (Supplementary Figs. 1 and 2). The BMY of the Alamo parent was 26% higher than the Kanlow parent at ETREC (Supplementary Fig. 1). At PREC, the BMY of Kanlow was 3% higher than Alamo (Supplementary Fig. 2). The PHT of Kanlow was 10 and 19 cm taller than Alamo at ETREC and PREC (Table 2; Supplementary Figs. 1 and 2).

### Linkage map and QTL mapping

**Fig. 2.**
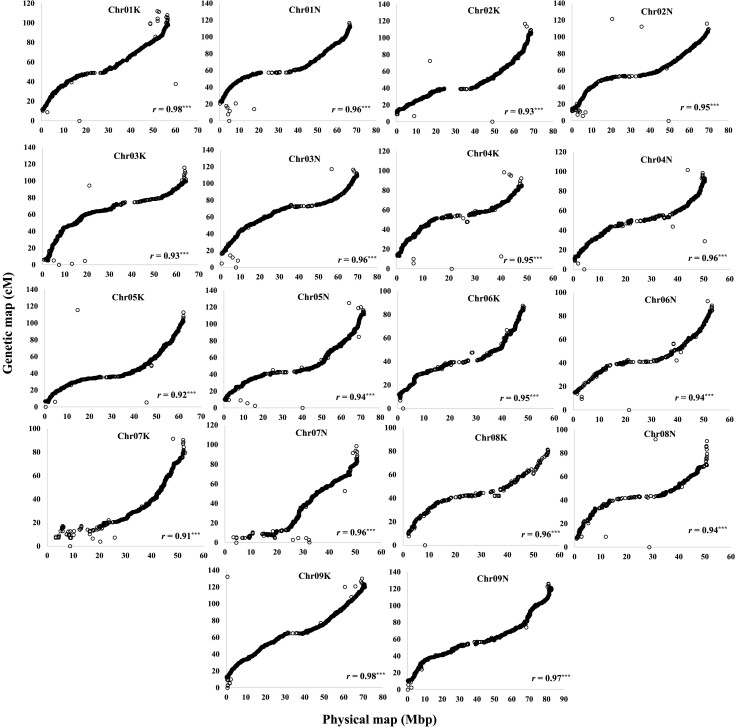
Relationships between physical map based on *Panicum virgatum* L. v5 and genetic map developed from 8 Alamo and Kanlow populations. The 18 linkage groups were formed using Lep-Map3. Each dot represents a single SNP marker. *r*, correlation coefficient between physical and genetic map.

A linkage map was constructed using 17,251 SNP markers, resulting in 18 linkage groups. The total map length was 1,941 cM (Fig. 2), and the average distance between markers was 0.11 cM. The correlation test indicated that the genetic map is highly correlated with the physical map position of the switchgrass reference genome (version 5.0) averaged across all chromosomes (*r* = 0.95) (Fig. 2). The highest correlation between the genetic map and the reference genome was observed on chromosome 1K and 9K (*r* = 0.98), whereas the least correlation was with chromosome 7K (*r* = 0.91). Among the mapped chromosomes, chromosome 9K possessed the greatest number of SNPs (1,582) with the largest map coverage (132 cM), while chromosome 8K possessed the least number of SNPs (403) and the chromosome 6K had the shortest map coverage (87 cM) (Fig. 2).

**Fig. 3.**
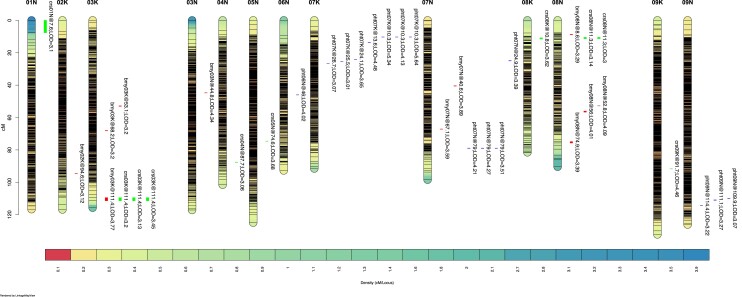
Identification of QTL for biomass yield (bmy), plant height (pht), and crown size (crs) detected on Alamo (female) and Kanlow (male) linkage map. Eighteen linkage groups (“K” and “N” sub-genomes) corresponding to 18 switchgrass chromosomes were formed using Lep-Map3 in which QTL detected on 13 chromosomes were presented in the figure. Significant QTL at *P* ≤ 0.05 are presented with their chromosomal position and LOD score. Each bar represents QTL position. The color bar represents the distribution density of the SNP marker per cM.

A total of 36 significant QTL peaks for BMY, PHT, and CRS were identified with LOD scores ranging from 3.0 to 5.6, and phenotypic variability explained (PVE) by QTL varied from 0.2 to 6.6% (Table 3; Fig. 3). Twenty-three QTL peaks showed negative additive effects, and 13 QTL peaks showed positive additive effects, indicating the presence of both additive and nonadditive effects of BMY, PHT, and CRS QTL on hybrid populations. Chromosomes 7K harbored a higher number of QTL (*n* = 7), followed by chromosomes 3K, 7N, and 8N (*n* = 6). Five QTL for BMY were identified across populations on chromosomes 2K, 3K, 3N, 7N, and 8N, respectively (Table 3; Fig. 3). The QTL for BMY (LOD 3.39 and 4.09) detected on chromosome 8N at ETREC, and PREC showed the highest PVE value (5.6% for each QTL) with positive additive effects (0.22 and 0.34) (Table 3). The minor effect QTL for BMY was detected on chromosome 3K across locations with a 2.5% PVE value and negative additive effect (−0.72) (Table 3). Four QTL for BMY (LOD 3.29–4.09) between 8.6 and 111.4 cM identified on chromosome 8N were significant in an individual and across environments and had a total of 18.1% PVE. Three QTL peaks for BMY (LOD 3.2–3.77) between 53.1 and 111.4 cM identified on chromosome 3K were significant across environments and expressed 7.7% PVE. In addition, 2 QTL peaks for BMY (LOD 3.59–3.89) between 40.6 and 67.1 cM were identified on chromosome 7N and expressed 7.7% PVE (Table 3). The presence of the QTL allele was associated with a significant increase in BMY from 0.5 to 1.8 Mg ha^−1^ at ETREC and by 1.6 Mg ha^−1^ at PREC (Table 3).

**Table 3.** Biomass yield, plant height, and crown size QTL identified on across Alamo and Kanlow populations for each location (ETREC and PREC) and year (2019, 2020, and 2021), and across environments (location and year).

Trait	LG	Marker name	Marker size (Mbp)	LOD	Position (cM)	AC*^a^*	AD*^a^*	BC*^a^*	BD*^a^*	PVE (%)	Additivity	Location_year*^b^*
Biomass yield (Mg ha^−1^)	02K	3462	66.8	3.12	94.6	3.7	4	3.5	3.4	3.79	0.12	ETREC_2019
	03K	6559	17.1	3.2	53.1	12.3	10.9	12.2	12	2.52	−0.72	Across locations 2020
	03K	6265	63.8	3.77	111.4	12.5	11.4	13	12.7	2.68	−0.58	Across locations 2021
	03K	6260	9.5	3.2	68.2	10.3	9.83	11.1	11.1	2.53	−0.32	Across locations years
	03N	7289	61.6	4.34	44.8	11.6	11.1	13.2	12.3	4.73	−0.26	ETREC_2020
	07N	14798	32.3	3.89	40.6	13.6	12	12.3	12.2	4.95	−0.79	ETREC_2021
	07N	14547	45.6	3.59	67.1	10.7	12.1	11.6	11	2.76	0.72	PREC_across years
	08N	15564	50.7	3.39	74.9	3.4	4	3.6	3.5	5.57	0.34	ETREC_2019
	08N	15658	40.0	4.09	52.8	10.8	11.2	9.6	9.6	5.56	0.22	PREC_2019
	08N	15973	1.2	3.29	8.6	11.6	12.8	11.7	11.4	2.68	0.65	Across locations 2020
	08N	15637	42.1	4.01	56.0	11.9	12.1	10.5	10.8	4.31	0.1	PREC_across years
Plant height (cm)	06N	13372	36.0	4.02	46.0	240.2	241.8	234.5	233.6	6.55	0.83	ETREC_2019
	07K	14366	17.4	4.48	13.6	232.3	224	226.2	225.3	3.04	−4.17	Across locations 2019
	07K	14388	16.3	5.34	10.3	250.4	240	242.8	241.9	3.71	−5.01	Across locations 2020
	07K	14388	16.3	4.13	10.3	248.1	236.7	240	239.7	3.25	−5.48	Across locations 2021
	07K	14259	30.9	3.01	25.5	234.2	228.1	224.9	230.1	3.86	−3.24	PREC_2020
	07K	14264	30.6	3.65	24.1	228.2	220.3	218.7	223.4	4.93	−4.3	PREC_2021
	07K	14388	16.3	5.64	10.3	244	234	237	235	3.65	−4.87	Across locations years
	07K	14231	31.2	3.07	26.7	227	221	219	222	4.06	−3.39	PREC_across years
	07N	14932	27.9	3.39	24.9	258.7	260.9	254.2	254.4	4.76	0.94	ETREC_2020
	07N	14447	49.4	4.21	79.0	225.2	235.5	229	226.8	6	5.07	PREC_2020
	07N	14447	49.4	4.27	79.0	219.4	228.3	220.8	220.3	4.94	4.45	PREC_2021
	07N	14447	49.4	3.51	79.0	218	228	222	221	5.32	4.71	PREC_across years
	09N	77	80.9	3.27	111.1	217.5	209.5	218.4	217.6	4.2	−3.83	PREC_2019
	09N	90	80.9	3.07	109.9	240	232	238	239	2.24	−4.08	Across locations years
	09N	44	81.5	3.22	114.4	246.2	238	244	245.8	2.3	−4.2	Across locations 2020
Crown size (0–5 score)	01N	2515	4.3	3.1	7.6	3.8	3.6	3.5	3.6	4.04	−0.12	ETREC_2021
	03K	6265	63.8	3.2	111.4	3.4	3.2	3.4	3.6	4.97	−0.14	PREC_2019
	03K	6265	63.8	3.13	111.4	3.5	3.3	3.4	3.6	4.19	−0.15	PREC_2020
	03K	6265	63.8	3.45	111.4	3.7	3.49	3.62	3.81	4.92	−0.14	PREC_across years
	04N	9021	49.7	3.06	87.7	3.5	3.8	3.7	3.7	3.73	0.13	ETREC_2020
	05N	11528	59.4	3.68	74.6	3	3.2	3.2	3.1	0.22	−0.78	Across locations 2019
	08K	15113	2.1	3.62	10.8	3.2	3.4	3.5	3.4	5.14	0.11	PREC_2019
	08N	15952	1.6	3.14	11.3	4	3.9	3.8	3.7	2.38	−0.01	Across locations 2021
	08N	15952	1.6	3	11.3	3.8	3.7	3.5	3.5	4.66	−0.07	ETREC_2021
	09K	17120	57.8	4.46	91.7	3.9	3.8	3.9	3.9	1.17	−0.1	Across locations 2021

LG, linkage group (“K” and “N” sub-genomes) corresponding to 18 switchgrass chromosomes; LOD, logarithm of the odds significant at *P* ≤ 0.05.

Biomass yield (Mg ha^−1^), plant height (cm), and crown size (1–5 score) associated with the QTL allele; AC, AD, BC, and BD, in which AB and CD alleles were associated with Kanlow and Alamo parents. The significant contribution of allele/s on QTL was identified using Tukey–Kramer HSD (*P* < 0.05); PVE, phenotypic variation (%) explained by QTL derived in the context of a composite interval mapping model (Broman and Sen 2009).

ETREC (Knoxville, TN), PREC (Crossville, TN), across locations (ETREC and PREC), and across years (2019, 2020, and 2021).

Four PHT QTL significant in at least one set of analyses were identified on chromosomes 6N, 7K, 7N, and 9N (Table 3; Fig. 3). The PHT QTL detected on chromosomes 6N (6.55%) and 7N (6%) showed the highest PVE values with positive additive effects (0.83 and 5.07) that were significant at ETREC and PREC (Table 3). The minor effect QTL for PHT detected on chromosome 9N across locations and years had a 2.2% PVE value with a −4.08 additive effect. Seven QTL peaks for PHT (LOD 3.01–5.64) identified between 10.3 and 26.7 cM on chromosome 7K were significant at PREC and across environments. Four QTL peaks for PHT (LOD 3.39–4.27) detected between 24.9 and 79.0 cM on chromosome 7N were significant in ETREC and PREC (Table 3). Three additional QTL peaks for PHT located between 109.9 and 114.4 cM on chromosome 9N were significant at PREC and across environments. The presence of the QTL allele was associated with the significant increase in PHT, up to 7 cm at ETREC and 9 cm at PREC (Table 3).

Seven significant QTL for CRS were identified in at least one location-year combination or across the location on chromosomes 1N, 3K, 4N, 5N, 8K, 8N, and 9K with PVE ranged from 0.2 to 5.1% (Table 3; Fig. 3). The CRS QTL detected on chromosome 8K had a higher PVE value (5.14%) and a positive additive effect (0.11). In contrast, the QTL detected on chromosome 5N had a smaller PVE value (0.22%) and a negative additive effect (−0.78) (Table 3). Three CRS QTL peaks were identified at 111.4 cM on chromosome 3K and were significant at PREC. Two other CRS QTL peaks located at 11.3 cM on chromosome 8N were significant at ETREC and across locations. The presence of QTL allele was associated with a significant increase in CRS, up to 9% at ETREC and 12% at PREC (Table 3).

The pleiotropic effect of QTL at the same or overlapping positions across environments was examined for each trait. Three PHT QTL peaks co-located on chromosome 7K (at 10.3 cM) were identified across environments (Table 3). Two additional PHT QTL peaks on chromosome 7N (at 79.0 cM) were detected at PREC in 2020 and 2021, and across years. Similarly, 3 CRS QTL peaks colocated on chromosome 3K (at 111.4 cM) were significant across environments. Furthermore, 2 CRS QTL peaks on chromosomes 8N (at 11.3 cM) were identified across and ETREC environments. The SNP marker, 6265, associated with QTL peaks for BMY and CRS identified on chromosome 3K (at 111.4 cM) were detected across environments (Table 3), which is also supported by the moderate association observed between CRS and BMY (*R^2^* = 0.45) across the environments in this study.

## Discussion

### Phenotypic variation

This study revealed transgressive phenotypic segregation in both directions for each trait at the 2 locations among the progeny of lowland parents. The effect of the year was significant for all evaluated traits at ETREC but only for the CRS at PREC (Table 1). Variations in plant performances across years, particularly at ETREC, could be associated with missing plots due to animal damage and harvesting errors. In addition, the transplanting date was delayed by 48 days at ETREC relative to PREC, and severe weed infestation was observed during the establishment year. This could have resulted in the poorer establishment and lower bud initiation rates in the late summer and fall of the establishment year at ETREC. The BMY was higher in the second and third years and lower in the first year of the postestablishment period in both locations (Supplementary Figs. 1 and 2). Newly transplanted seedlings have poor canopy establishment in the first year. Other switchgrass field studies have demonstrated similar effects in the establishment period (Alexopoulou *et al*. 2008; Bhandari *et al*. 2017).

Other environmental factors, such as precipitation and temperature, can also partially explain the reduction in BMY at ETREC compared to PREC. The annual precipitation during 2019, 2020, and 2021 were 165, 183, and 140 cm at ETREC and 194, 184, and 156 cm at PREC. The total precipitation was 15.6 cm less at ETREC than at PREC across the studied years. The precipitation at ETREC was 24 cm less in 2021 and 18 cm higher in 2020 than in 2019. However, during active growth in June through August 2020, plants were stressed due to low total precipitation (29.4 cm) as compared to 2019 (34.5 cm) and 2021 (37.8 cm), and this could have affected on detection of environment-sensitive QTL for BMY. In addition to low precipitation, ETREC was warmer and dryer than PREC during the study period. The average temperature at ETREC was 15.2 °C compared to 12.8 °C at PREC, likely leading to combined water and heat stress (Shrestha *et al*. 2021). Poudel *et al.* (2019a) found that lowland switchgrass exhibits cold tolerance and can develop cultivars with high BMY potential suitable for growing in northern latitudes. In contrast to BMY, PHT at ETREC was taller than at PREC. The cooler temperature at PREC could have affected the trait's full expression, resulting in shorter plants than at ETREC.

As results reported herein, transgressive segregation has been reported in BMY, vegetative growth, PHT, and flowering traits in switchgrass hybrid populations derived from lowland AP13 and upland VS16 genotypes (Serba *et al.* 2015; Ali *et al.* 2019). Shrestha *et al*. (2021) found high mid-parent and high-parent BMY heterosis in the same populations assessed in this study which they hypothesized could have resulted from the recombination of complementary favorable alleles from parents.

Taller PHT and higher CRS were associated with higher BMY in our study. Other studies have also identified the secondary traits, including PHT, spring green-up, and vegetative growth traits, as important contributors to higher BMY (Serba *et al.* 2015; Ali *et al.* 2019). The conventional method of selecting these traits is challenging and costly because they typically require several years of field assessment. Therefore, an alternative method of selection using molecular markers is important to speed up the selection process.

### Linkage map and QTL mapping

The linkage map produced using Lep-MAP3 in this study agrees well with previously published genetic maps in switchgrass produced using several different marker technologies and parental genotypes (Okada *et al.* 2010; Lowry *et al.* 2015; Serba *et al*. 2015; Ali *et al*. 2019; Poudel *et al.* 2019b). The advantages of using Lep-MAP3 were that it could handle a large quantity of data in a memory-efficient manner and integrate data from multiple full-sib families. Furthermore, given genotype probabilities, it could impute genotypes and easily output phased AB × CD data for 2 heterozygous parents.

Our study identified a major effect QTL for BMY on chromosome 8N at 52.8 and 74.9 cM. The other QTL reported in this chromosome was by Ali *et al*. (2019), who identified a QTL for spring green-up at 60.35 cM, days to flowering at 49.98 cM, and vegetative growth period at 44.96 cM in the AP13 (lowland) × VS16 (upland) full-sib population. Genomic regions associated with BMY, vegetative growth period, and days to flower have been identified on chromosomes 2K, 3K, 3N, and 7N (Serba *et al*. 2015; Ali *et al*. 2019). The QTL for BMY identified on chromosome 2K at 94.6 cM is close to another QTL reported for days to flower at 91.6 cM by Ali *et al*. (2019). The BMY QTL identified on chromosome 3K at 53.1, 68.2, and 111.4 cM were 0.6, 4.9, and 10.2 cM away from the BMY QTL reported by Serba *et al.* (2015). These QTL (on chromosome 3K) had negative additive effects, which were also in agreement with the QTL effects reported by Serba *et al*. (2015).

This study identified a major QTL for PHT (LOD 6.26) with high PVE (7%) on chromosome 6N, which was specific to the environment and exhibited a positive additive effect (0.83). Ali *et al*. (2019) identified a spring green-up QTL on chromosome 6N, which was located 1.1 cM away from the major QTL for PHT identified through our study. Seven PHT QTL peaks on chromosome 7K (10.3–26.7 cM) and 4 peaks on 7N (24.9–79.0 cM) were identified in our study. Serba *et al.* (2015) identified 4 PHT QTL peaks on chromosome 7K using the female linkage map in which one of the QTL (at 25.6 cM) was located 0.1–1.5 cM distant from 3 PHT QTL identified in our study. Three QTL peaks for PHT were identified on chromosome 9N across environments. Another study (Lowry *et al*. 2015) identified a PHT QTL on the same chromosome, which they found colocalized with the major BMY QTL.

CRS is a postharvest direct visual assessment of crown biomass that represents a combination of tillering ability, tiller density, and stem thickness. Due to the simple scoring method in field settings, positive correlation with BMY, and the possibility of remote data acquisition, CRS would be valuable to assess in breeding nurseries where spaced planting is practiced. Seven CRS QTL were identified on chromosomes 1N, 3K, 4N, 5N, 8K, 8N, and 9K, which relates indirectly to other tiller measurements in the literature. The CRS QTL identified on chromosome 8K had the largest PVE value (5.1%), followed by QTL on chromosomes 3K and 8N. Lowry *et al*. (2015) identified QTL for tiller length, tiller width, tiller mass, tiller angle, flag leaf width, leaf area, and postharvest regrowth on chromosomes 1N, 5N, 8 K, and 8N in a full-sib mapping population derived from a cross between Alamo and Kanlow. Chang *et al*. (2016) identified QTL for tillering ability, tiller diameter, tiller DW, node number per tiller, plant base size, and plant girth on chromosomes 1N, 3K, and 9K in the hybrid populations derived a cross of 2 lowland switchgrass genotypes. Another study (Ali *et al*. 2019) reported a spring green-up QTL on chromosome 9K, which was 5.6 cM away from the CRS QTL identified in our study.

Two peaks for the BMY QTL identified on chromosomes 8N shifted by 3.2 cM across years. The PHT QTL peaks identified on chromosome 7K shifted 1.2–3.3 cM across the years. Three additional PHT peaks on chromosome 9N shifted up to 4.5 cM. Serba *et al*. (2015) have also reported slight shifts in QTL positions for BMY due to significant QTL and environment interactions. They indicated that the differences in environmental factors, including drought and soil types, could produce variability in QTL detection between the 2 locations (Serba *et al*. 2015). In addition, the variability herein was also across years, which could be affected by both environmental factors and the age of plants. Plot management was similar over the years, but the plant stands were 2-, 3-and, 4-years old in 2019, 2020, and 2021. As switchgrass produces higher biomass at later postestablishment periods than in the early postestablishment period (Bhandari *et al*. 2017), this could lead to variations in the QTL detection. Environment-sensitive QTL has been reported for vegetative growth, flowering date, PHT, and surface wax in switchgrass (Serba *et al.* 2015; Ali *et al.* 2019; Bragg *et al*. 2020), while in sorghum fresh panicle weight, tiller number per plant, and flowering also displayed this responsiveness (Shiringani *et al.* 2010).

Six of the 7 QTL for BMY, PHT, and CRS identified at ETREC were mapped to the “N” sub-genome. Compared to ETREC, the same number of QTL were mapped in both the “K” and “N” sub-genomes at PREC. Major effect QTL for BMY (on chromosome 8N) and PHT (6N and 7N) were also mapped on the N sub-genome. It provides some support for the observation that sub-genome dominance (as described by Lovell *et al.* 2021) plays a role in the adaptive evolution of switchgrass.

### Conclusions

The presence of genetic variation for BMY, PHT, and CRS in F1 families derived from intercrosses of Alamo and Kanlow lowland parents enabled the detection of QTL associated with these traits. The presence of favorable allelic combinations increased BMY up to 1.8 Mg ha^−1^, PHT up to 9 cm, and CRS up to 12% across populations. The markers 15658 and 15564, associated with BMY QTL on chromosome 8N, exhibited high PVE (5.6%). Furthermore, the QTL for BMY and CRS were found colocated on chromosome 3K, by which the selection imposed on one trait simultaneously improves the other trait. Markers associated with the QTL have important implications for marker-assisted breeding. These results indicate that the QTL identified across multiple segregating populations could be transferrable across populations. The QTL markers for the traits evaluated can be utilized to capitalize on the intra- and intercultivar genetic variation to develop new and improved switchgrass cultivars.

## Supplementary Material

jkad061_Supplementary_Data

## Data Availability

The phenotypic and genotypic data of each individual used in the QTL analysis are available at the Dryad digital repository (https://doi.org/10.5061/dryad.hmgqnk9mm; Shrestha *et al.* 2023). There are 5 files in total. The “File 1” contains phenotype data for each genotype and parents evaluated at PREC and ETREC from 2019 to 2021. “File 2” has the genotype name, library index, total reads, bases, and Q30%. The “File 3” has SNP ID numbers, SNP locations on chromosomes, map positions, and SNP scores. The cross was used as a 4-way cross for QTL analysis (Xu 1996), where the male parent Kanlow (K) was assigned as “1,” and the female parent Alamo (A) was assigned as “2.” The phased output data from the 4-way cross, i.e. 11, 12, 21, and 22, were represented by AC, BC, AD, and BD, respectively (“File 3”). The Progeny file and linkage map are presented in “Files 4 and 5.” The authors affirm that all data necessary to confirm the article's conclusions are present in the article, figures, and tables. Supplemental material available at G3 online.
