# Supplementary material for: Mapping quantitative trait loci for biomass yield and yield-related traits in lowland switchgrass (Panicum virgatum L.) multiple populations
Source: G3 (Bethesda). 2023 Mar 22;13(5):jkad061. doi: 10.1093/g3journal/jkad061 (PMC10151402; doi:10.1093/g3journal/jkad061)
Supplement: jkad061_Supplementary_Data [file jkad061_supplementary_data.zip › Figure_S1_G3-2023-404164.docx]

Figure S1 Mean distribution for biomass yield (a), plant height (b), and crown size (c) of six individual and one combined populations at ETREC (East Research and Education Center, Knoxville, Tennessee) in 2019, 2020, and 2021. 12A-261 × 12K-245 and 12A-263 × 12K-250 were consolidated into one cross based on segregation analysis. ‘A’ indicates the Alamo source parent, and ‘K’ indicates the Kanlow source parent.
