# Supplementary material for: Mapping quantitative trait loci for biomass yield and yield-related traits in lowland switchgrass (Panicum virgatum L.) multiple populations
Source: G3 (Bethesda). 2023 Mar 22;13(5):jkad061. doi: 10.1093/g3journal/jkad061 (PMC10151402; doi:10.1093/g3journal/jkad061)
Supplement: jkad061_Supplementary_Data [file jkad061_supplementary_data.zip › Figure_S2_G3-2023-404164.docx]

Figure S2 Mean distribution for biomass yield (a), plant height (b), and crown size (c) of seven individual and one combined population at PREC (Plateau Research and Education Center, Knoxville, Tennessee) in 2019, 2020, and 2021. 12A-261 × 12K-245 and 12A-263 × 12K-250 were consolidated into one population based on segregation analysis. ‘A’ indicates the Alamo source parent, and ‘K’ indicates the Kanlow source parent. ‘TN1304-08’ and ‘TN13009-08’ are selections from Alamo and Kanlow parents.
