# Supplementary material for: Mapping quantitative trait loci for biomass yield and yield-related traits in lowland switchgrass (Panicum virgatum L.) multiple populations
Source: G3 (Bethesda). 2023 Mar 22;13(5):jkad061. doi: 10.1093/g3journal/jkad061 (PMC10151402; doi:10.1093/g3journal/jkad061)
Supplement: jkad061_Supplementary_Data [file jkad061_supplementary_data.zip › Table_S1_G3-2023-404164.docx]

**Table S1. Summary of filtering steps used for SNPs and individuals.**

| **Filtering steps** | **Number** |
| --- | --- |
| Initial number of variant sites | 4678008 |
| Sites remaining after setting sites with a depth of one to missing and filtering sites with >10% missing data | 226318 |
| Sites informative in "1" or more families | 153320 |
| Sites informative in "6" or more families | 24684 |
| Sites remaining after pruning redundant sites | 17358 |
| Sites remaining after clustering and manual filtering out mis-grouped markers | 17251 |
| Initial number of individuals | 788 |
| Remaining number of individuals after the removal of selfed individuals | 754 |
| Remaining number of individuals after the removal of problematic individuals with too many recombinations | 648 |
